# Application of the strip clear-cutting system in a running bamboo (*Phyllostachys glauca* McClure) forest: feasibility and sustainability assessments

**DOI:** 10.3389/fpls.2024.1335250

**Published:** 2024-02-12

**Authors:** Kuan Liang, Guangru Wang, Zhan Shen, Juan Wu, Na Zou, Hongying Yu, Shebao Yu, Fusheng Chen, Jianmin Shi

**Affiliations:** ^1^ College of Forestry, Jiangxi Agricultural University, Nanchang, China; ^2^ Key Laboratory of National Forestry and Grassland Administration on Forest Ecosystem Protection and Restoration of Poyang Lake Watershed, Jiangxi Agricultural University, Nanchang, China; ^3^ CAS Key Laboratory of Forest Ecology and Management, Institute of Applied Ecology, Chinese Academy of Sciences, Shenyang, China; ^4^ Jiangxi Provincial Key Laboratory of Soil and Water Conservation, Jiangxi Academy of Water Science and Engineering, Nanchang, China

**Keywords:** bamboo forest, economic benefit, membership value, productivity trait, sustainability

## Abstract

**Introduction:**

As a renewable forest resource, bamboo plays a role in sustainable forest development. However, traditional cutting systems, selection cutting (SeC) and clear-cutting (ClC), result in an unsustainable production of bamboo forests due to labor-consuming or bamboo degradation. Recently, a strip clear-cutting (StC) was theoretically proposed to promote the sustainability of bamboo production, while little is known about its application consequence.

**Methods:**

Based on a 6-year experiment, we applied the strip clear-cutting system in a typical running bamboo (*Phyllostachys glauca* McClure) forest to assess its feasibility and sustainability. Using SeC and ClC as controls, we set three treatments with different strip widths (5 m, 10 m, and 20 m) for strip clear-cutting, simplified as StC-5, StC-10, and StC-20, respectively. Then, we investigated leaf physiological traits, bamboo size and productivity, population features, and economic benefits for all treatments.

**Results:**

The stands managed by StC had high eco-physiological activities, such as net photosynthetic rate (*P*_n_), photosynthetic nitrogen use efficiency (PNUE), and photosynthetic phosphorus use efficiency (PPUE), and thus grew well, achieved a large diameter at breast height (DBH), and were tall. The stand biomass of StC (8.78 t hm^-2^ yea^r-1^) was 1.19-fold and 1.49-fold greater than that of SeC and ClC, respectively, and StC-10 and StC-20 were significantly higher than SeC or ClC (*p*< 0.05). The income and profit increased with the increase in stand density and biomass, and StC-20 and StC-10 were significantly higher than SeC or ClC (*p*< 0.05). Using principal components analysis and subordinate function analysis, we constructed a composite index to indicate the sustainability of bamboo forests. For the sustainability assessment, StC-10 had the highest productive sustainability (0.59 ± 0.06) and the second highest economic sustainability (0.59 ± 0.11) in all cutting treatments. StC-10 had the maximum overall sustainability, with a value of 0.53 ± 0.02, which was significantly higher than that of ClC (*p*< 0.05).

**Conclusion:**

The results verified that StC for *Phyllostachys glauca* forests is feasible and sustainable as its sustainability index outweighs those of traditional cutting systems (SeC and ClC), and 10 m is the optimum distance for the strip width of StC. Our findings provide a new cutting system for managing other running bamboo forests sustainably.

## Introduction

Forest sustainability is crucial to human well-being, and sustainable forest management plays a vital role in sustainable development (Singh et al., 2009; MacDicken, 2016; Viccaro and Caniani, 2019). Productive and economic sustainability are key assessment components of sustainable forest management (Hansmann et al., 2012; Holden et al., 2014; Brukas et al., 2015; Bartniczak and Raszkowski, 2018; Viccaro and Caniani, 2019). Productive sustainability requires forest management that does not weaken forest productivity (Bartniczak and Raszkowski, 2018; Viccaro and Caniani, 2019). Economic sustainability refers to maintaining economic income over time without compromising sustainability (Foy, 1990; Brukas et al., 2015). Generally, productive and economic sustainability emphasize different aspects of sustainability but are indispensable components of sustainable development (Holden et al., 2014; Brukas et al., 2015).

Although natural forest area decreases by approximately 6.5 million hectares every year (MacDicken, 2016; Viccaro and Caniani, 2019), the bamboo forest is known as an increasing forest, with a total area of more than 30 million hectares in China (Du et al., 2018; Kang et al., 2022). As clonal plants, bamboo species are fast-growing and have a short renewable cycle. Thus, bamboo is a good substitute for wood and plays a role in satisfying forestry production needs (Guo et al., 2013). However, traditional cutting systems (selection cutting and clear-cutting) for bamboo forests are either labor-consuming or degrade the bamboo, which limits the sustainable development of bamboo forests.

A cutting system is an important way to achieve high productivity and economic sustainability in bamboo forests. At present, the cutting systems for bamboo forests are selection cutting and clear-cutting (Wang et al., 2016; Mao et al., 2017; Liu and Hu, 2018). Selection cutting can achieve high productive sustainability by selectively cutting down a certain proportion of mature plants every cutting season to keep bamboo forests in a rational age structure (Liu and Hu, 2018). However, the productivity advantage comes at the expense of high labor costs and low economic profit (Wang et al., 2016; Mao et al., 2017; Liu and Hu, 2018). By contrast, clear-cutting saves cutting costs by cutting down all plants in a cutting area every cutting season. In addition, clear-cutting is liable to result in a degradation of bamboo forests as no existing mature plants supply nutrients to newborn bamboo to support its growth (Liu and Hu, 2018). Therefore, the low cost of clear-cutting is at the expense of productive sustainability. As selection cutting and clear-cutting have evident drawbacks in economic sustainability or productive sustainability, it is urgent to develop a sustainable cutting system for managing bamboo forests more sustainably.

**Figure 1:**
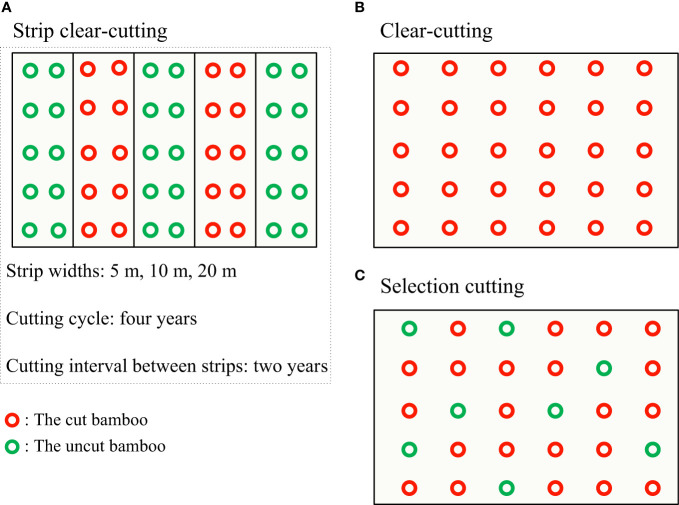
The design of cutting systems in *Phyllostachys glauca* bamboo forests. **(A)** The strip clear-cutting system with different strip widths (5 m, 10 m, and 20 m); at least nine strips were established for each width. **(B)** The clear-cutting system. **(C)** The selection cutting system.

Strip clear-cutting refers to applying clear-cutting in forest strips along the direction of the slope and retaining uncut patches in cut areas (Ocaña-Vidal, 1992; Picchio et al., 2018). Strip clear-cutting is a sustainable cutting method for timber extraction (Rondon et al., 2010) but it is seldom applied to bamboo forests. Considering the source-sink characteristic of clonal integration (Marshall, 1996; Kleinhenz and Midmore, 2001; Shi et al., 2021), the strip clear-cutting system could be a potential solution for the sustainable management of bamboo forests because the newborn bamboo in clear-cut strips could obtain nutrients from the uncut strips to support their regeneration and then avoid degradation (Wang et al., 2016). After a partial clear-cutting experiment in stands of a typical running bamboo (*Phyllostachys glauca*) was carried out, the maximum distance of clonal integration was 5 m because the new shoots in the clear-cutting area were significantly smaller than those in the uncut area once they were more than 5 m away from the uncut side (Wang et al., 2016). Thus, a strip clear-cutting system protocol was proposed for running bamboo forests (see **Figure 1A**), i.e., dividing a bamboo stand into equally wide strips along a slope, clear-cutting every other strip, and then clear-cutting the “uncut” strips over a certain cycle (e.g. every 2 years), and so on (Wang et al., 2016). This protocol combines the merits of clear-cutting (saving labor and cost) and selection cutting (sustainable production). Although the strip clear-cutting system is arguably feasible and sustainable in theory, we still do not know its consequences in practice. Furthermore, an optimum strip width for this cutting system needs to be determined.

*Phyllostachys glauca* McClure, a woody and evergreen bamboo, is a typical running bamboo native to China. The bamboo forms pure forests and covers a large area in Ruichang City. In this study, using traditional cutting systems (selection cutting and clear-cutting) as controls and setting strip clear-cutting with different widths, we aimed to verify the productive and economic sustainability of bamboo forest managed by a strip clear-cutting system and determine a suitable strip width through a long-term trial with *Phyllostachys glauca* forest in Ruichang City. Based on the preliminary results, we hypothesized that: (1) strip clear-cutting has advantages in productive and economic sustainability over selection cutting and clear-cutting because it incorporates the merits of the two traditional cutting systems, and (2) as the maximum distance of nutrient supply observed in the field cutting experiment was 5 m and a distance less than 5 m is not convenient for cutting (most culms were approximately 5 m long), a strip width of 5 m is the best option for the strip clear-cutting system in practice.

## Materials and methods

### Species and site description

*Phyllostachys glauca* is widely distributed from the Yellow River Valley to the Yangtze River Valley. It is 5-12 m in height and 20-50 mm in diameter, and has excellent economic value, e.g., shoots for food and culms for building and ornaments (Wang et al., 2016; Shi et al., 2021). As an economic species, *Phyllostachys glauca* brings considerable benefits to local people.

The study site was located in Ruichang City (29°23’06″ N-29°51’11″ N, 115°06’31″ E-115°43’45″ E), Jiangxi province, China, where the largest area of natural *Phyllostachys glauca* forest in China grows, with a total area of 9 938 hm^2^ (Wang et al., 2016; Shi et al., 2021; Wu et al., 2022). In the local area, *Phyllostachys glauca* usually forms pure stands with few understory species. Ruichang City has a subtropical humid monsoon climate that receives 1394 mm precipitation annually, with an average temperature of 16.6°C.

### Traditional cutting systems for *Phyllostachys glauca*

The bamboo forests were predominantly managed by the clear-cutting system with a cutting cycle of 3 years, and the bamboo population could not fully recover under this short cycle of clear-cutting. Consequently, this long-term cutting leads to a progressive decline in bamboo stands and even flowering. Another cutting system, selection cutting with a cutting cycle of 3 or 4 years, was only applied to a small proportion of bamboo forests because of labor consumption. Owing to high stand density, approximately 90% of individuals were cut for operating convenience. The two cutting systems barely maintained sustainable forest production.

### Experiment design

#### Strip clear-cutting system design

We used the protocol proposed by Wang et al. (2016), which was described as aforementioned, to design the strip clear-cutting system. Strip width and cutting cycle, two key parameters of the cutting system, were designed as follows. Three strip widths were set to detect a suitable distance for bamboo growth and recovery, which were 5 m, 10 m, and 20 m. As the selection cutting system with a cutting cycle of 3 or 4 years can maintain the bamboo forest healthily in the local area, we designed a 4-year cutting cycle for the strip clear-cutting system in this study. In practice, a bamboo stand was divided into strips of equal width (5 m, 10 m, or 20 m) along the hill slope and continuously numbered from number one. Then, all even strips were clear-cut first, and the uncut strips (odd strips) would be clear-cut 2 years later. Accordingly, the even and odd strips were alternately clear-cut every 2 years (**Figure 1A**).

#### Cutting experiment design

In December 2016, bamboo forests located at the same elevation, slope aspect, degree and position were selected to develop the cutting experiments. Ten plots of 10 m × 10 m were surveyed for each kind of bamboo stand, and the results showed that they were similar in the diameter at breast height (DBH), plant height, and stand density (**Table 1**). Taking selection cutting and clear-cutting systems as controls, the strip clear-cutting system was designed as three treatments, which were strip clear-cutting with strip widths of 5 m (StC-5), 10 m (StC-10), and 20 m (StC-20) (**Figure 1**). The experiment design was a balanced incomplete block design due to terrain constraints. For the strip clear-cutting experiment, at least nine strips were set for each treatment, and the strip lengths ranged from 50 to 100 m. Four 5 m × 5 m plots in StC-5, six 5 m × 10 m plots in StC-10, and five 5 m × 10 m plots in StC-20 were established in the cut strips and uncut strips. Clear-cutting was alternatively carried out for the initial cut and uncut strips in December 2018 and 2020. The selection cutting (SeC) and clear-cutting (ClC) experiments were carried out in a stand at least 50 m wide and 100 m long. Five and four 5 m × 10 m plots in SeC and ClC were randomly selected, respectively. Selection cutting and clear-cutting systems were both carried out every 3 years.

**Table 1:** The initial characteristics of bamboo forests before cutting.

Bamboo stands	Diameter at breast height (cm)	Plant height (m)	Stand density (Plants hm^-2^)
Strip clear-cutting	2.02 ± 0.12a	4.36 ± 0.24a	36819 ± 5003a
Selection cutting	2.05 ± 0.16a	4.40 ± 0.31a	38555 ± 6588a
Clear-cutting	2.02 ± 0.11a	4.35 ± 0.22a	35361 ± 5582a

Data are means ± S.E (n =10). The same lowercase letters indicate non-significant differences among different bamboo stands (p > 0.05).

### Methods

#### Plot survey

In December 2016, the DBH and height of each individual were measured in each plot before bamboo cutting. From 2017 to 2021, the DBH, plant height, and number of newborn bamboos in each plot were investigated every year.

#### Leaf physiological traits

The net photosynthetic rate (*P*_n_) of different treatments was measured using an open gas exchange system with a red and blue light source (LI-6400; LiCor Inc., Lincoln, NE, USA), and the measurements were conducted from 9:00 h to 11:00 h on sunny days in August. Three mature sun leaves on healthy individual plants (five plants in each plot) were randomly selected and measured at a CO_2_ concentration of 400 μmol mol^−1^, photosynthetic photon flux density of 1500 mmol m^−2^ s^−1^, and flow rate of 500 mmol s^-1^. When the measurement parameters were stable, three values were recorded every 10 s for each leaf, and the average value of each leaf was used for further analysis.

To determine the specific leaf area (SLA), 50 mature leaves in each plot were collected, and their leaf areas were measured using a CI-203 Portable Laser Area Meter (CID Inc., Camas, WA, USA). Afterwards, the leaves were oven-dried at 60°C for 48 h and the dry mass was determined. Then dried leaf samples were ground into a fine powder for chemical analysis. Leaf phosphorous (P) and nitrogen (N) concentrations were determined using a discontinuous chemical analyzer (Cleverchem 200+, DeOem-Tech GmbH, Hamburg, Germany) after digestion with sulfuric acid (H_2_SO_4_).

#### Economical investigation

The most economical part of *Phyllostachys glauca* is the culms, which are widely used as the holder for vegetable cultivation, garden building, flagpoles, and so on. The sale price of this bamboo culm depends on the culm length. We investigated the local sale price of culms and the cutting cost from 2017 to 2021. As the bamboo forests grow naturally and the culms are sold *in situ*, the management cost of those bamboo forests is only the labor costs of cutting. On a hectare basis, the profit of bamboo forests was calculated as the income (the product of culm yield and sale price) subtracted from the cutting cost. Here, the extra cutting cost for strip clear-cutting set the boundary lines between strips at the first cutting operation.

### Indicator calculation

#### Productivity indicators

Stand density was calculated as the total number of bamboos divided by plot area. Individual biomass was calculated using the quadratic model proposed by Zou et al. (2020), which used DBH and height as predictors to estimate biomass. Then, the stand biomass was the sum of the total individual biomass in a plot. Eventually, the annual stand density and annual stand biomass were calculated as stand density and biomass divided by the cutting cycle.

The calculations of the evenness and uniformity of bamboo forests followed the methods of Zheng and Hong (1998). Specifically, evenness was calculated as stand density divided by the standard deviation of subplot density, and uniformity was calculated as average DBH divided by the standard deviation of subplot average DBH. In addition, the recruitment rate was estimated as the ratio between the number of newborn bamboos and the number of pre-existing bamboos in a plot.

SLA (cm^2^ g^-1^) was calculated by dividing leaf area by leaf dry mass, and photosynthetic nitrogen use efficiency (PNUE) and photosynthetic phosphorus use efficiency (PPUE) were calculated by dividing *P*_n_ by leaf N and P concentrations, respectively.

#### Economic indicators

Bamboo wood production (BWP) refers to the biomass of bamboo culms. Based on our investigation, the sale price of bamboo culms was classified into seven classes according to their height (**Table 2**). The sale income was the sum of all the bamboo individuals in a plot with their specific heights and prices, as shown in Equation 1:

**Table 2:** Averaged sale prices of *Phyllostachys glauca* culms in Ruichang City from 2017 to 2021.

Plant height (m)	2.5≤H<3	3≤H<4	4≤H<5	5≤H<6	6≤H<7	7≤H<8	H≥8
Averaged price (¥ plant^-1^)	0.209	0.400	0.567	0.850	1.067	1.367	1.850


(1) $$I=\sum_{i=1}^{x}(R_{i}\times n_{i})/A/C$$

where *I* is the income (¥ hm^-2^ year^-1^); $R_{i}$ and $n_{i}$ are the sale price and the number of bamboos in a height grade in a plot, respectively; *A* is the plot area; and *C* is the cutting cycle, i.e., 6 years for selection cutting, clear-cutting, and strip clear-cutting. The number and height of culms in a plot were investigated during the cutting operation. Similar to the calculation of income, cutting costs were also calculated in the unit of ¥ hm^-2^ year^-1^, i.e., cutting costs divided by stand area and cutting cycle. The return on investment (ROI) represents the ratio of cost to income.

#### Sustainability calculation

Using principal components analysis (PCA) and subordinate function analysis (SFA), we constructed a comprehensive index based on multiple indicators to indicate the sustainability of bamboo forests, which was calculated using Equation 2 (Fu et al., 2004; Mrosek et al., 2006; Shi et al., 2009; Balana et al., 2010):


(2) $$SI=\sum_{i=1}^{n}\left(W_{i}\times S_{i}\right)$$

where *SI* is the sustainability index, $W_{i}$ is the weight vector of the *i* sustainability indicator, and $S_{i}$ is the membership value of the *i* sustainability indicator.

The weight of sustainability indicators ( $W_{i}$ ) was determined by PCA. Based on the cumulative percentage of principal sustainability components and the component capacity score coefficient values, $W_{i}$ was calculated using Equation 3:


(3) $$W_{i}=C_{i}/\sum_{i=1}^{n}C_{i}$$

where $\;C_{i}$ is the component capacity score coefficient of the *i* sustainability indicator. The membership value ( $S_{i}$ ) was calculated as either ascending or descending functions (Equations 4, 5). An ascending function was used for a sustainability indicator with a positive component capacity score, and vice versa.


(4) $$S_{i}=\frac{\left(x_{ij}-x_{imin}\right)}{\left(x_{imax}-x_{imin}\right)}$$

(5) $$S_{i}=\frac{\left(x_{imax}-x_{ij}\right)}{(x_{imax}-x_{imin)}}$$

where $x_{ij}$ is the *j*-th observation value of the *i*-th sustainability indicator, and $x_{imax}$ and $x_{imin}$ are the maximum and minimum of the *i*-th sustainability indicator, respectively.

We defined three sustainability indexes, i.e., the productive sustainability index (*SI*_pr_), economic sustainability index (*SI*_ec_), and overall sustainability index (*SI*_o_). *SI*_pr_ was composed of 13 indicators: DBH, plant height, stand density, stand biomass, recruitment rate, evenness, uniformity, *P*_n_, PPUE, PNUE, leaf phosphorus concentration (LP), leaf nitrogen concentration (LN), and SLA. *SI*_ec_ was composed of five indicators: bamboo wood production, income, cost, return on investment (ROI), and profit. Then, *SI*_o_ was calculated using all the above 18 indicators.

### Statistics analysis

One-way ANOVA with Duncan’s multiple range test was used to examine the significance of the effect of the cutting on leaf physiological traits (*P*_n_, PPUE, PNUE, LP, LN, and SLA), bamboo size and productivity (DBH, plant height, stand density, and stand biomass), population features (recruitment rate, evenness, and uniformity), economic benefits (bamboo wood production, income, cost, ROI, and profit), and sustainability (*SI*_pr_, *SI*_ec_, and *SI*_o_). Pearson correlation analysis was performed to detect the relationships between physio-productivity traits, bamboo size and productivity, population features, economic benefits, and sustainability. The weight vector of each indicator was determined by principal component analysis (PCA). Redundancy analysis (RDA) was used to explore the relationships between sustainability indexes and their predictors (productivity traits and economic features). RDA was performed using CANOCO 5.0 (Microcomputer Power Corporation, USA). PCA and other statistical analyses were performed by SPSS 17.0 (SPSS Inc., Chicago, IL, USA), and figures were graphed with Origin Pro 8.5 (Origin Lab Corporation, Northampton, MA, USA).

## Results

### The productivity traits of different cutting treatments

#### Physio-productivity traits

**Figure 2:**
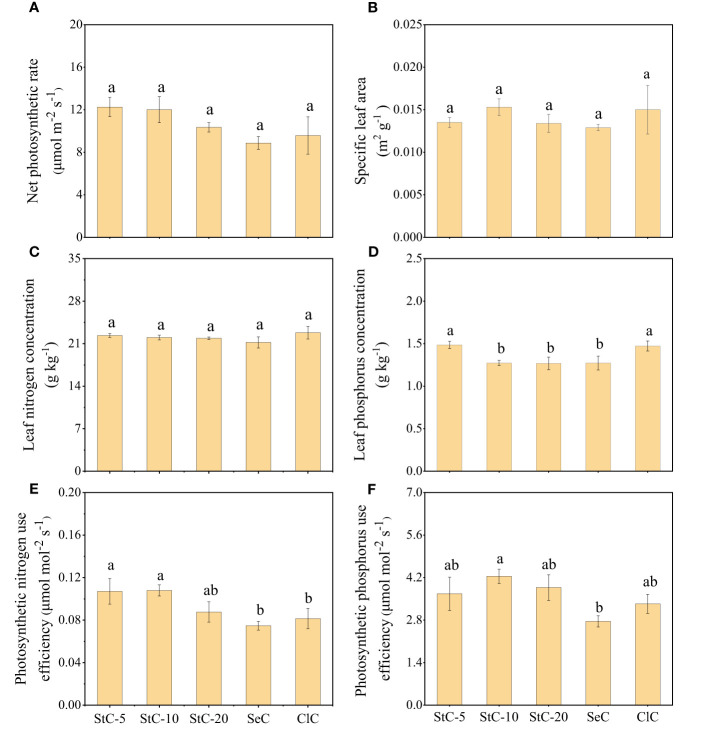
The effect of cutting systems on leaf physiological traits. **(A)** Net photosynthetic rate. **(B)** Specific leaf area. **(C)** Leaf nitrogen concentration. **(D)** Leaf phosphorus concentration. **(E)** Photosynthetic nitrogen use efficiency. **(F)** Photosynthetic phosphorus use efficiency. Data are means ± S.E. StC-5, strip clear-cutting of 5 m width treatment, n = 4; StC-10, strip clear-cutting of 10 m width treatment, n = 6; StC-20, strip clear-cutting of 20 m width treatment, n = 5; SeC, selection cutting treatment, n = 5; ClC, clear-cutting treatment, n = 4. The small letters indicate significant differences between different treatments in the same indicator (*p*< 0.05).

The net photosynthetic rate (*P*_n_), specific leaf area (SLA), leaf nitrogen concentration (LN), leaf phosphorus concentration (LP), photosynthetic phosphorus use efficiency (PNUE), and photosynthetic phosphorus use efficiency (PPUE) were varied in bamboo stands with different cutting systems (**Figure 2**). *P*_n_ and SLA were similar in different cutting treatments (*p* > 0.05), while their maximum and minimum values both occurred in StC-10 and SeC treatments, respectively (**Figures 2A, B**). As for LN, all treatments were similar (*p* > 0.05) (**Figure 2C**). However, the LP values of StC-5 and ClC were 1.49 ± 0.04 g kg^-1^ and 1.47 ± 0.06 g kg^-1^, respectively, and significantly higher than that of other treatments (*p*< 0.05) (**Figure 2D**). The strip clear-cutting treatments (StC-5, StC-10 and StC-20) were higher than the treatments of SeC and ClC in PNUE and PPUE (**Figures 2E, F**). PNUE and PPUE of StC-10 (0.11 ± 0.01 μmol mol^-2^ s^-1^ and 4.11 ± 0.22 μmol mol^-2^ s^-1^) were significantly higher than those of SeC (*p*< 0.05).

#### Bamboo forest productivity

**Figure 3:**
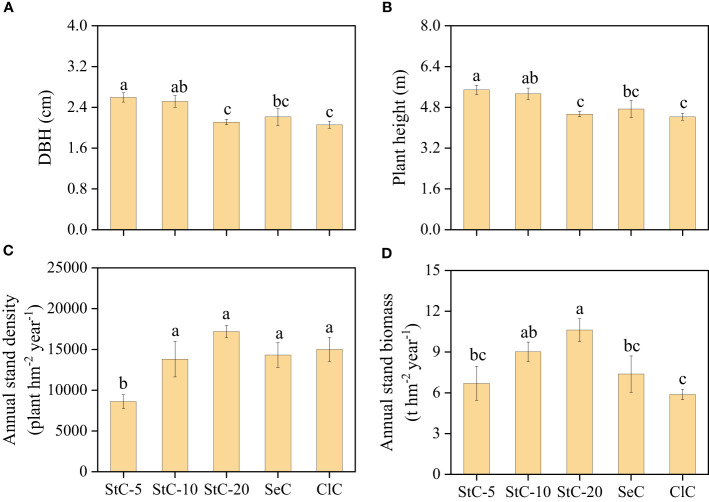
The effect of cutting systems on the bamboo growth of *Phyllostachys glauca*. **(A)** DBH. **(B)** Plant height. **(C)** Annual stand density. **(D)** Annual stand biomass. Data are means ± S.E. DBH, diameter at breast height; StC-5, strip clear-cutting of 5 m width treatment, *n* = 4; StC-10, strip clear-cutting of 10 m width treatment, *n* = 6; StC-20, strip clear-cutting of 20 m width treatment, *n* = 5; SeC, selection cutting treatment, *n* = 5; ClC, clear-cutting treatment, *n* = 4. The small letters indicate significant differences between different treatments in the same indicator (*p*< 0.05).

Different cutting systems have imposed an evident influence on bamboo size (DBH and plant height), stand density, and productivity (stand biomass) (**Figure 3**). In strip clear-cutting treatments, the bamboo sizes decreased with an increase in strip width, and the bamboos in StC-5 and StC-10 were similar in size (*p* > 0.05) but larger than those in other treatments. On the contrary, the stand density of the strip clear-cutting treatments increased with an increase in strip width, and the stand density of StC-20 had the highest value (17198.52 ± 750.65 plant hm^-2^ year^-1^) in all treatments. Owing to its relatively large size and high stand density, StC-20 had the greatest stand biomass (10.62 ± 0.84 t hm^-2^ year^-1^), which was 1.58-, 1.18-, 1.43-, and 1.81-fold greater than StC-5, StC-10, SeC, and ClC, respectively.

#### Population features

**Figure 4:**
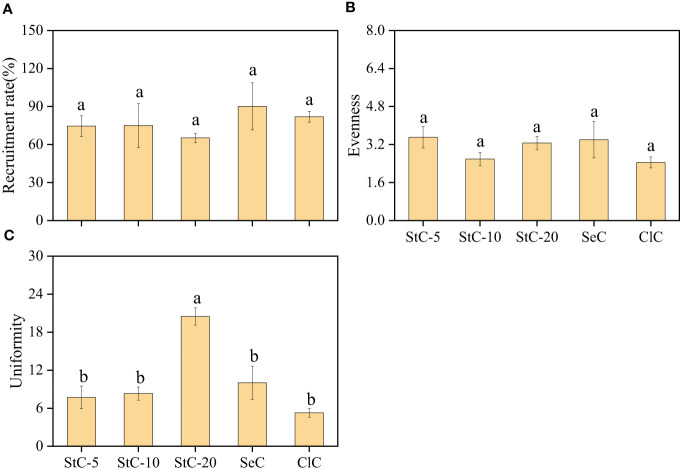
The effect of different cutting systems on population features of *Phyllostachys glauca* stands. **(A)** Recruitment rate. **(B)** Evenness. **(C)** Uniformity. Data are means ± S.E. StC-5, 5 m strip clear-cutting, *n* = 4; StC-10, 10 m strip clear-cutting, *n* = 6; StC-20, 20 m strip clear-cutting, *n* = 5; SeC, selection cutting, *n* = 5; ClC, clear-cutting, *n* = 4. The small letters indicate significant differences between different treatments of the same indicator (*p*< 0.05).

There were no significant differences in recruitment rate and evenness between bamboo stands managed by different cutting systems (*p* > 0.05) (**Figure 4**). Among the cutting treatments, StC-20 and ClC were the lowest in recruitment rate and evenness, which were 65.20 ± 3.64% and 2.45 ± 0.23, respectively. Conversely, the stand uniformity of StC-20 was significantly higher than other cutting treatments (*p*< 0.05), with a value of 20.50 ± 1.36.

### The economic benefits of different cutting treatments

**Figure 5:**
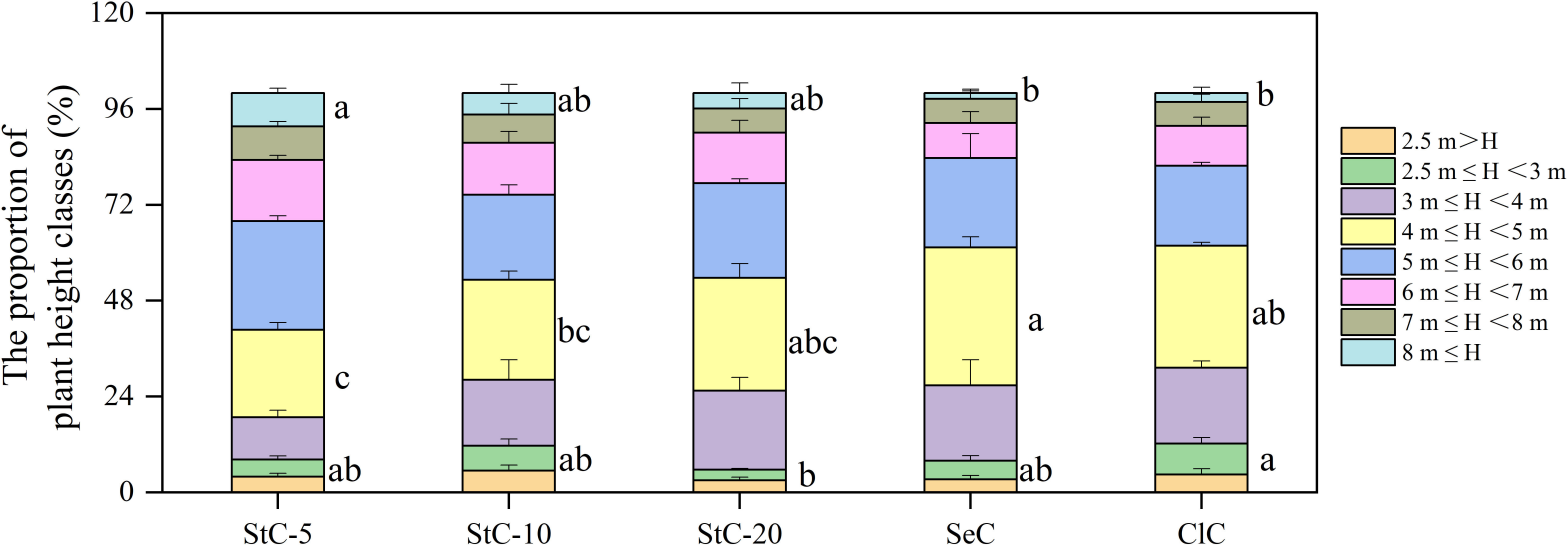
The proportion of plant height classes in bamboo stands treated by different cutting systems. Data are means ± S.E. StC-5, strip clear-cutting of 5 m width treatment, *n* = 4; StC-10, strip clear-cutting of 10 m width treatment, *n* = 6, StC-20, strip clear-cutting of 20 m width treatment, *n* = 5; SeC, selection cutting treatment, *n* = 5; ClC, clear-cutting treatment, *n* = 4. The small letters indicate significant differences between different treatments in the same indicator (*p*< 0.05).

The bamboo stands under different cutting systems showed a distinct structure of height classes (**Figure 5**), which determined a specific sale price of culms (as shown in **Table 2**). The proportions of plant heights over 5 m in strip clear-cutting stands were 59.27%, 46.76%, and 46.31% for StC-5, StC-10, and StC-20, respectively, which decreased with an increase of strip width and were all higher than SeC (38.67%) and ClC (38.23%). Compared with SeC and ClC, the strip clear-cutting treatments showed a relatively lower proportion of the height (H) class of 4m ≤ H< 5 m and a relatively higher proportion of the height class of H ≥ 8 m. As a result, the bamboo stands managed by strip clear-cutting systems, especially StC-5 and StC-10, had a higher proportion of long culms than SeC and ClC.

**Figure 6:**
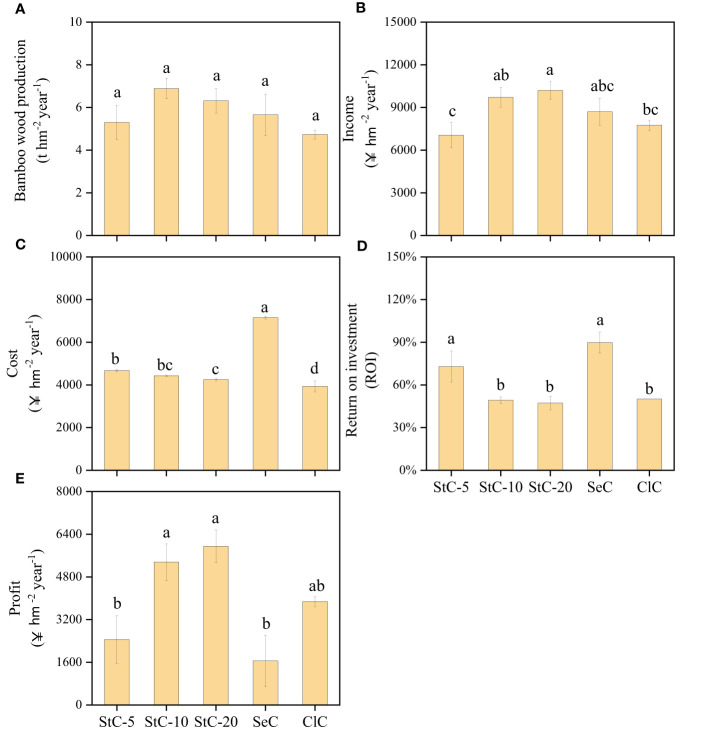
The economic statistics of bamboo stands under different **(A)** Bamboo wood production. **(B)** Income. **(C)** Cost. **(D)** Return on investment. **(E)** Profit. Data are means ± S.E. StC-5, strip clear-cutting of 5 m width treatment, *n* = 4; StC-10, strip clear-cutting of 10 m width treatment, *n* = 6; StC-20, strip clear-cutting of 20 m width treatment, *n* = 5; SeC, selection cutting treatment, *n* = 5; ClC, clear-cutting treatment, *n* = 4. The small letters indicate significant differences between different treatments in the same indicator (*p*< 0.05).

Different cutting systems have a significant influence on bamboo economics (**Figure 6**, *p*< 0.05). StC-10 obtained the greatest bamboo wood production (6.89 ± 0.48 t hm^-2^ year^-1^), which was 1.30-, 1.09-, 1.22- and 1.45-fold greater than that of StC-5, StC-20, SeC, and ClC, respectively. With long culms and a high stand density, the bamboo stands managed by StC-10 and StC-20 obtained higher incomes than those managed by SeC and ClC. The income of StC-20 was as high as 10, 194 yuan hm^-2^ year^-1^, which was 1.45-, 1.05-, 1.17- and 1.32-fold greater than that of StC-5, StC-10, SeC, and ClC, respectively. The cutting cost was highest in SeC and lowest in ClC, which were 7, 152 yuan hm^-2^ year^-1^ and 3, 934 yuan hm^-2^ year^-1^, respectively. Additionally, the cutting cost of strip clear-cutting treatments ranged from 4, 248 yuan hm^-2^ year^-1^ to 4, 671 yuan hm^-2^ year^-1^. In terms of the return on investment (ROI), SeC and StC-5 were significantly higher than StC-10, StC-20, and ClC. As a result, StC-20 and StC-10 obtained a higher profit (5, 957 yuan hm^-2^ year^-1^ and 5, 352 yuan hm^-2^ year^-1^) than other treatments.

### Sustainability indicators and their principal contributors

#### Sustainability indicators

**Figure 7:**
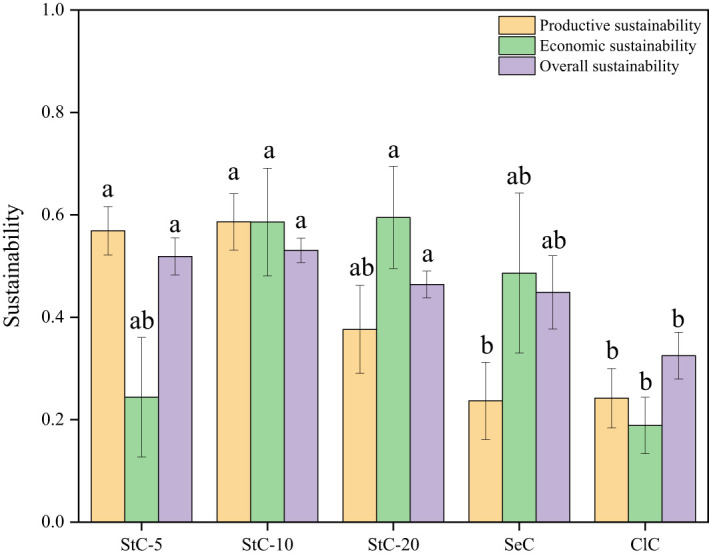
The sustainability indicators of bamboo stands under different cutting systems. Values are means ± S.E, StC-5: strip clear-cutting of 5 m width treatment, *n* = 4; StC-10: strip clear-cutting of 10 m width treatment, *n* = 6; StC-20, strip clear-cutting of 20 m width treatment, *n* = 5; SeC: selection cutting treatment, *n* = 5; ClC: clear-cutting treatment, *n* = 4. Different small letters indicate the significant differences between different treatments in the same indicator (*p*< 0.05).

The sustainability indicators, including productive sustainability, economic sustainability, and overall sustainability, varied for the bamboo stands under different cutting systems (**Figure 7**). As for productive sustainability, StC-5 and StC-10 were significantly higher than other treatments (*p*< 0.05), with values of 0.57 ± 0.05 and 0.59 ± 0.06, respectively. StC-20 and StC-10 had higher economic sustainability values (0.60 ± 0.10 and 0.59 ± 0.11), which were 3.16-fold and 3.11-fold greater than ClC, respectively. Combining the productivity and economic aspects, the overall sustainability of strip clear-cutting treatments was higher than SeC and ClC, and StC-10 obtained the greatest value of 0.53 ± 0.02 among all treatments. Generally, compared with selection cutting and clear-cutting, strip clear-cutting models exhibited good sustainability in this bamboo forest management.

#### Principal contributors to the sustainability indicators

**Figure 8:**
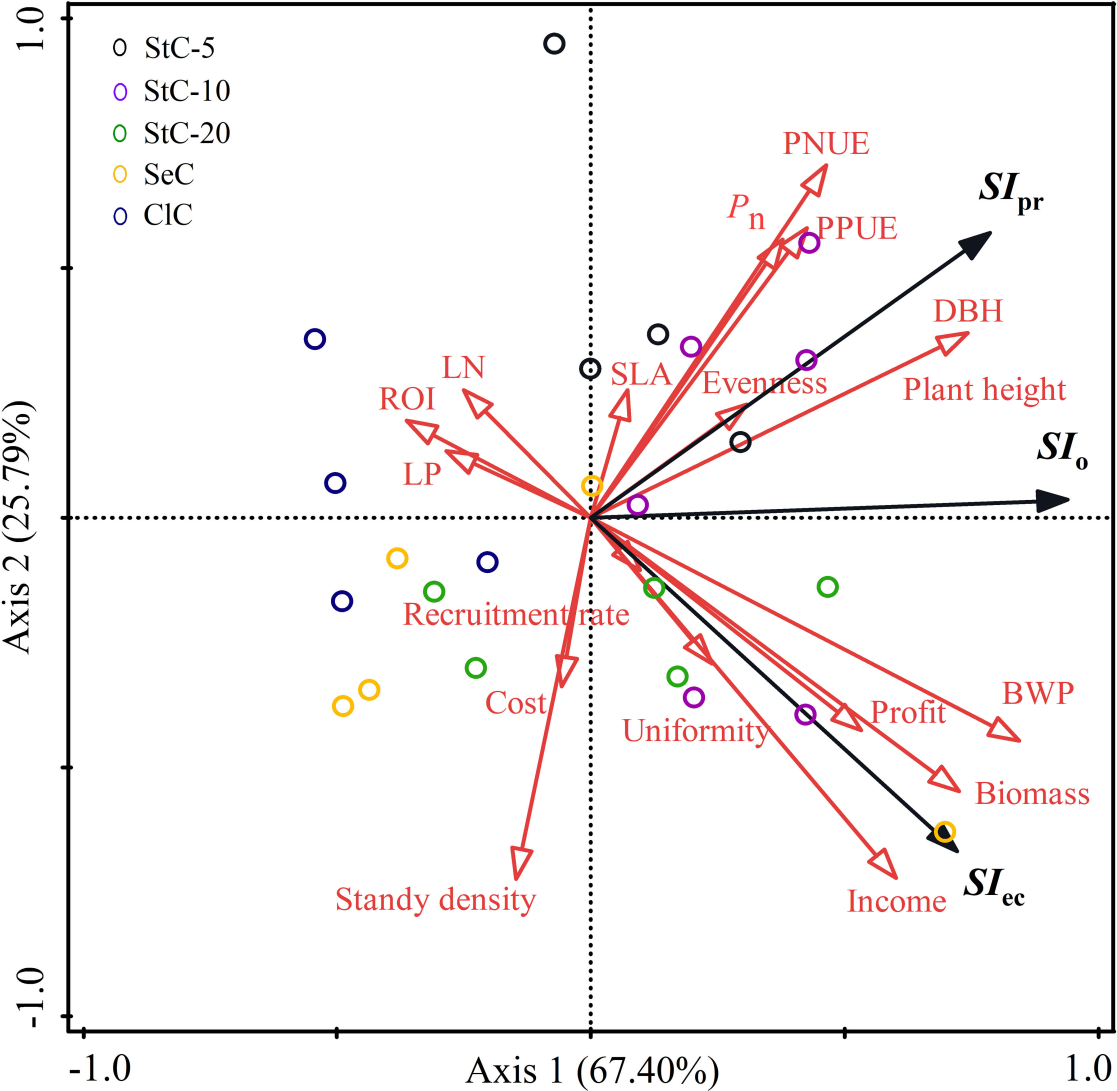
Redundancy analysis (RDA) of productivity traits, economic features, and sustainability indexes of *Phyllostachys glauca* stands managed by different cutting systems. Black arrows, response variables; red arrows, explaining variables. StC-5, strip clear-cutting of 5 m width treatment; StC-10, strip clear-cutting of 10 m width treatment; StC-20, strip clear-cutting of 20 m width treatment; SeC, selection cutting treatment; ClC, clear-cutting treatment. *SI*_pr_, productive sustainability index; *SI_ec_*, economic sustainability index; *SI*_o_, overall sustainability index; *P*_n_, net photosynthetic rate; SLA, specific leaf area; LN, leaf nitrogen concentration; LP, leaf phosphorus concentration; PNUE, photosynthetic nitrogen use efficiency; PPUE, photosynthetic phosphorus use efficiency; DBH, the diameter at breast height; BWP, bamboo wood production; ROI, return on investment.

The redundancy analysis (RDA) showed the relationships between productivity traits, economic features, and sustainability indexes of *Phyllostachys glauca* stands managed by different cutting systems (**Figure 8**). As sustainability indexes were calculated with the component capacity score coefficient of each indicator (see Equation 3), the six indicators (PNUE, PPUE, DBH, plant height, *P*_n_, and stand density) closely related to productive sustainability were the principal contributors of *SI*_pr_, with a negative contribution of stand density and a positive contribution from the other five indicators. Since other predictors such as uniformity, evenness, LP, LN, SLA, and recruitment rate weakly correlated to productive sustainability, they had limited contributions. Likewise, the bamboo wood production, income, return on investment, and profit of bamboo stands were four principal contributors to economic sustainability because they were closely associated with *SI*_ec_. When all predictors were included, the overall sustainability was mainly determined by plant height, DBH, bamboo wood production, stand biomass, evenness, and income. However, income and bamboo wood production were mostly determined by the productivity indicators (stand biomass and density), with significant correlation coefficients (*p*< 0.01) of 0.92 (income and stand biomass), 0.61 (income and stand density) and 0.91 (bamboo production and stand biomass), respectively. Furthermore, stand density was significantly and negatively associated with DBH (*r* = -0.70, *p*< 0.001) and plant height (*r* = -0.70, *p*< 0.001), while bamboo wood production was significantly and positively associated with DBH (*r* = 0.53, *p*< 0.01) and plant height (*r* = 0.53, *p*< 0.01). Thus, the bamboo individual size not only affected productive sustainability but also governed economic sustainability, and it was the key factor influencing the overall sustainability of bamboo stands.

## Discussion

### The feasibility of applying the strip clear-cutting system in *Phyllostachys glauca* forest

Our first hypothesis that strip clear-cutting has advantages in productive and economic sustainability over selection cutting and clear-cutting because it incorporates the merits of the two traditional cutting systems, was mainly supported. Productive, economic, and overall sustainability of strip clear-cutting were higher than SeC and ClC except for the economic sustainability of StC-5 (**Figure 7**).

The bamboo stands managed by strip clear-cutting systems had larger individual plants (DBH and plant height) and a greater averaged stand biomass than those managed by SeC and ClC (**Figure 3**). As plant size and stand biomass were the main contributors to productive sustainability, the treatments of strip clear-cutting outweighed the treatments of SeC and ClC in productive sustainability. Our results were consistent with previous studies, which indicated that the strip cutting of *Phyllostachys edulis* led to greater DBH, plant height, and biomass than selection cutting and clear-cutting (Zhang et al., 2020). This was likely explained by the high eco-physiological activities of the bamboo under strip clear-cutting because *P*_n_, PNUE, and PPUE are important factors that promote plant growth by increasing photosynthates and the plant nitrogen utilization rate, especially in the case of nutrient deprivation (Ghannoum et al., 2005; Zhong et al., 2019; Mugo et al., 2021; Nasar et al., 2021). A higher PUNE resulted in a higher crop nitrogen utilization rate and thus enhanced crop yield (Ghannoum et al., 2005; Zhao et al., 2013; Mugo et al., 2021). In this study, we also detected strong correlations between *P*_n_ and PNUE, and PNUE and bamboo size, which in turn affected biomass (**Figure 8**).

Moreover, bamboos are clonal plants and have the trait of clonal integration, which can translocate resources (photosynthate, mineral nutrients, water, etc.) between ramets connected with rhizomes along a source-sink gradient (Pitelka and Ashmun, 1985; Alpert, 1991; Price et al., 1996; Dong et al., 2019; Shi et al., 2022). As for the strip clear-cutting system, bamboos in the uncut strip were source ramets and could provide resources to sink ramets (newborn bamboos) in the cut strip to support their growth (D’hertefeldt and Jónsdóttir, 1994; Chen et al., 2015; Wang et al., 2016; Shi et al., 2021; Zheng et al., 2022). A higher *P*_n_ leads to more photosynthate production, which could be translocated and utilised by the connected bamboo ramets and then enhance the final yield (Li et al., 2016; Li et al., 2018). Therefore, we inferred that the high photosynthetic capacity and clonal integration among bamboos increased individual size and consequently led to a greater stand biomass in strip clear-cutting stands than in clear-cutting stands, in which no source ramets supply nutrients to the newborn bamboos as the bamboo plants were all cut. Unlike the case of applying SeC in Moso bamboo (*Phyllostachys edulis*) stands, the bamboo stands managed by selection cutting in this study did not show a competitive advantage in sustainable production. The reason that selective cutting maintains good sustainable productivity in the Moso bamboo forest lies in the low cutting intensity because few mature individuals were cut, with a proportion of less than one-quarter of the total. However, in our case, the average stand density of *Phyllostachys glauca* was as high as 3.7 plants m^-2^. For the sake of operating convenience, the local farmers cut approximately 90% of individuals in a cutting cycle, and the few individuals kept supplied limited nutrients for bamboo regeneration, resulting in lower productive sustainability than SeC.

In addition to good productivity, the bamboo stands managed by strip clear-cutting also have good economic sustainability. The economic indicators of bamboo wood production and income were highly related to stand density and biomass, which were mainly determined by individual bamboo size (**Figure 8**). Although the cutting cost of strip clear-cutting was higher than that of clear-cutting (Wang et al., 2016; Liu and Hu, 2018), StC-10 and StC-20 outcompeted SeC and ClC in economic sustainability due to their higher stand density and plant height. On the contrary, StC-5 had lower economic sustainability than other treatments because it had the lowest stand density (**Figure 3C**). As a clonal plant, ramets of *Phyllostachys glauca* are analogous to twigs of trees. Thus, bamboo cutting is similar to tree pruning, and more intensive pruning leads to more regeneration of new shoots (Zhou et al., 2009; Zheng et al., 2022; Zhou et al., 2022). In this study, the low stand density of StC-5 was attributed to the weakest cutting strength in all treatments.

Compared with traditional cutting systems (SeC and ClC), the strip clear-cutting system is efficient and sustainable in bamboo forest management and thus has advantages over productivity and economy. Additionally, some studies confirmed that the application of clear-cutting in bamboo forests led to the degradation of bamboo resources (Tan et al., 2017; Liu and Hu, 2018). Therefore, our results indicated that the strip clear-cutting system is feasible in bamboo forest management.

### An appropriate strip width for the strip clear-cutting system for *Phyllostachys glauca* forest

Our second hypothesis that a strip width of 5 m is the best option for the strip clear-cutting system in practice was not supported. According to the values of the overall sustainability index, the best strip width is 10 m instead of 5 m (**Figure 7**). The productive sustainability of StC-10 was similar to StC-5, and the economic sustainability of StC-10 was the second highest. StC-10 outcompeted other cutting treatments (StC-5, StC-20, SeC, and ClC) in overall sustainability.

The 10-m strip width of strip clear-cutting has the maximum productive sustainability in all treatments because it has advantages in traits closely related to productive sustainability, such as PNUE, PPUE, DBH, plant height, and stand density (**Figure 8**). In the strip clear-cutting treatments, StC-10 had higher SLA, PNUE, and PPUE than StC-5 and StC-20 (**Figure 2**). This indicated that the bamboo of StC-10 had stronger photosynthetic ability and resource-utilizing efficiency than StC-5 and StC-20 and increased bamboo growth. Likewise, previous studies found that a higher photosynthetic capacity leads to more photosynthate accumulation to accelerate the growth of *Phyllostachys edulis* (Li et al., 2018; Zheng et al., 2022). Furthermore, nutrient supply to the bamboo in the cut strips decreased with increasing strip width. Many studies confirmed that the strength of clonal integration (resource translocation between bamboos) decreased with an increase in the distance between source and sink ramets (Matlack, 1997; Stuefer et al., 2004; Shi et al., 2021), which was also supported by our results. Bamboo size decreased with the increase in strip width, i.e., the clonal integration distance (**Figures 3A, B**). Bamboo plants in cut strips need to receive nutrients from the bamboo in uncut strips on both sides to support their growth, and a shorter distance means more nutrient supplies. Field cutting and isotopic tracing (N^15^) experiments both suggested that the effective distance of clonal integration in *Phyllostachys glauca* is less than 5 m (Wang et al., 2016; Shi et al., 2021). Thus, it explains the large size of bamboo individuals in StC-5. As for StC-10, bamboo in cut strips could obtain nutrients from bamboo on either the left or right side of uncut strips no more than 5 m wide, which could result in a similar individual size to that of StC-5. Similarly, previous studies on the strip clear-cutting of Moso bamboo also found that individual sizes with strip widths of 3 m and 6 m were larger than with strip widths of 9 m and the SeC and ClC treatments (Tan et al., 2017), and the DBH of new bamboo gradually decreased with the increase in strip width (Zhou et al., 2022). Furthermore, we found that stand density increased with increasing strip width. Thus, bigger individual size and higher stand density lead to greater stand biomass of StC-10.

With a moderate stand density and large bamboo, the 10 m strip width of strip clear-cutting also has good economic sustainability. More individuals and long culms in a stand result in higher income and profit. StC-10 and StC-20 achieved significantly higher economic sustainability than ClC (*p*< 0.05). Although StC-20 had the greatest economic sustainability (0.60 ± 0.10), it had lower productive sustainability than StC-5 and StC-10, which were only 66.14% and 64.15% of StC-5 and StC-10, respectively (*p*< 0.05). As the clear-cutting width (20 m) was longer than the effective distance of clonal integration (< 5 m), the newborn ramets in the central area of the cut strips could not receive any nutrients from the source ramets in the uncut strips on either side (Matlack, 1997; Stuefer et al., 2004; Shi et al., 2021). Thus, bamboo stands would be liable to be degraded by long-term management with StC-20, the same situation caused by ClC. Conversely, the stands managed by StC-5 produced fewer bamboo individuals than StC-10 and StC-20 and then obtained an economic sustainability (0.24 ± 0.12) of less than 40% of StC-10 and StC-20. Thus, a strip width of 10 m rather than 5 m was the best option in the forest management of *Phyllostachys glauca*.

### The potential application of the strip clear-cutting system for running bamboo management

*Phyllostachys glauca* is a running bamboo species that places ramets in new micro-habitats via the underground running rhizomes (Shi et al., 2021; Wang et al., 2023). For running bamboos, ramets connected with a rhizome can translocate their resource, such as water and nutrients (clonal integration), along a source-sink gradient (Shi et al., 2021; Shi et al., 2022). Strip clear-cutting produces a source-sink gradient between the cut strips and uncut strips, and the strip width determines the strength of clonal integration, which supports the growth of newborn ramets. Theoretically, the strip clear-cutting system suits other running bamboo species because they have the same clonal structure. To date, the strip clear-cutting system has only been used in two running bamboo species, *Phyllostachys glauca* and *Phyllostachys edulis* (Wang et al., 2016; Zhang et al., 2020; Zheng et al., 2022). Testing of the effect of different strip widths on bamboo regeneration showed that 10 m and 6 m were suitable strip widths for the cutting of *Phyllostachys glauca* and *Phyllostachys edulis* (Wang et al., 2016; Zhang et al., 2020; Zheng et al., 2022), respectively. However, there are 178 running bamboo species widely distributed in China (Gu et al., 2021). Most of those species are of high economic and productive value. Using an efficient and sustainable cutting system is a vital issue in increasing the management sustainability of bamboo forests. Therefore, the strip clear-cutting system deserves to be applied to other running bamboo species.

Although some studies developed strip clear-cutting in Moso bamboo forests in recent years, most of them focused on regeneration growth, soil traits, etc., rather than an assessment of management sustainability (Tan et al., 2017; Zhang et al., 2020; Zheng et al., 2022; Zhou et al., 2022). In this study, we evaluated the sustainability of *Phyllostachys glauca* forests under the strip clear-cutting system based on a 6-year field experiment. Thus, the long-term observation, across a complete strip clear-cutting cycle, offers a reliable and accurate assessment of this new cutting system. Our results have verified the feasibility and sustainability of applying the 10-m strip width of strip clear-cutting in *Phyllostachys glauca* forests. It provides a new option for managing running bamboo forests in a sustainable way.

## Conclusions

With traditional cutting systems for *Phyllostachys glauca* forests (selection cutting and clear-cutting), it is hard to achieve good sustainability in both productivity and economy. Based on a 6-year experiment, we assessed the feasibility and sustainability of strip clear-cutting, a new cutting system for bamboo stands, by comparing its productivity traits, economic features, and sustainability index with those of traditional cutting systems. The strip clear-cutting outweighed selection cutting and clear-cutting in productive and economic sustainability because it possessed the cutting convenience of clear-cutting and good nutrient supply for regeneration of selection cutting. Of the different strip widths, a 10 m wide strip of strip clear-cutting obtained the highest overall sustainability and is the optimum option for the strip clear-cutting system in practice. The results verified that strip clear-cutting with a 10-m strip width in *Phyllostachys glauca* forests is feasible and sustainable. Our findings provide a novel system for the cutting of other running bamboos in a sustainable way.

## Data availability statement

The original contributions presented in the study are included in the article/supplementary material. Further inquiries can be directed to the corresponding authors.

## Author contributions

KL: Investigation, Formal analysis, Writing – original draft, Writing – review & editing. GW: Investigation, Writing – review & editing. ZS: Formal analysis, Writing – review & editing. JW: Investigation, Writing – review & editing. NZ: Formal analysis, Writing – review & editing. HY: Writing – review & editing. SY: Conceptualization, Investigation, Writing – review & editing. FC: Writing – review & editing. JS: Conceptualization, Funding acquisition, Supervision, Writing – original draft, Writing – review & editing.
